# *Flowering Locus C (FLC)* Is a Potential Major Regulator of Glucosinolate Content across Developmental Stages of *Aethionema arabicum* (Brassicaceae)

**DOI:** 10.3389/fpls.2017.00876

**Published:** 2017-05-26

**Authors:** Setareh Mohammadin, Thu-Phuong Nguyen, Marco S. van Weij, Michael Reichelt, Michael E. Schranz

**Affiliations:** ^1^Biosystematics, Plant Sciences Group, Wageningen University and ResearchWageningen, Netherlands; ^2^Department of Biochemistry, Max Planck Institute for Chemical EcologyJena, Germany

**Keywords:** glucosinolates, development, QTL, Brassicaceae, *Aethionema*, multi-trait analyses

## Abstract

The biochemical defense of plants can change during their life-cycle and impact herbivore feeding and plant fitness. The annual species *Aethionema arabicum* is part of the sister clade to all other Brassicaceae. Hence, it holds a phylogenetically important position for studying crucifer trait evolution. Glucosinolates (GS) are essentially Brassicales-specific metabolites involved in plant defense. Using two *Ae. arabicum* accessions (TUR and CYP) we identify substantial differences in glucosinolate profiles and quantities between lines, tissues and developmental stages. We find tissue specific side-chain modifications in aliphatic GS: methylthioalkyl in leaves, methylsulfinylalkyl in fruits, and methylsulfonylalkyl in seeds. We also find large differences in absolute glucosinolate content between the two accessions (up to 10-fold in fruits) that suggest a regulatory factor is involved that is not part of the quintessential glucosinolate biosynthetic pathway. Consistent with this hypothesis, we identified a single major multi-trait quantitative trait locus controlling total GS concentration across tissues in a recombinant inbred line population derived from TUR and CYP. With fine-mapping, we narrowed the interval to a 58 kb region containing 15 genes, but lacking any known GS biosynthetic genes. The interval contains homologs of both the sulfate transporter *SULTR2;1* and *FLOWERING LOCUS C*. Both loci have diverse functions controlling plant physiological and developmental processes and thus are potential candidates regulating glucosinolate variation across the life-cycle of *Aethionema*. Future work will investigate changes in gene expression of the candidates genes, the effects of GS variation on insect herbivores and the trade-offs between defense and reproduction.

## Introduction

Plant fitness depends on a plants ability to reach the next generation. Thus, plants must be able to defend themselves from herbivores and pathogens throughout their life-cycle; first during vegetative growth, then at the time of flowering and finally during the production of fruits and seeds. Pest pressure and effects on survival and fitness can differ greatly across the growth of the plant (Van Zandt, 2007). Therefore, defensive compound quality and quantity can shift and be modified during various developmental stages (Brown et al., 2003). Also, there can be negative allocation and/or constitutive costs between plant defense and plant fitness (Manzaneda et al., 2010). Glucosinolates (GS, i.e., mustard oils) and their associated myrosinase enzymes form a two-component chemical plant defense in the Brassicales, defending against herbivores and pathogens. GS are nitrogen and sulfur-rich plant metabolites that hydrolyse, upon contact with the myrosinase enzyme, to form the herbivore-deterrent compounds nitriles and isothiocyanates (Halkier and Gershenzon, 2006; Sønderby et al., 2010). GS are spatially separated from myrosinase; hence the toxic compounds are only formed after an herbivore attack when the cell is ruptured (Koroleva et al., 2000). The GS biosynthesis additionally has links to fundamental biochemical and developmental processes such as auxin biosynthesis. The Brassicaceae specific IAOX auxin pathway shares intermediate compounds with the indolic GS pathway (Mano and Nemoto, 2012). Moreover GS can also be perceived by specialist herbivores as oviposition stimuli (Hopkins et al., 2009). Although all Brassicales contain GS, the highest diversity (of 120 different) GS compounds is found within the economically important family Brassicaceae (Halkier and Gershenzon, 2006; Edger et al., 2015). This diversity is thought to be due to a combination of gene and genome duplications within Brassicaceae and due to the selective pressure from co-adapting Brassicaceae Pierideae herbivores (Edger et al., 2015).

*Arabidopsis thaliana* is used as an important system to understand the molecular pathways underlying GS biosynthesis and flowering time. GS quality and quantity change throughout the development of *A. thaliana* (Petersen et al., 2002; Brown et al., 2003) and are influenced by the presence of nutrients, such as sulfur, that are incorporated into GS (Falk et al., 2007; Aarabi et al., 2016). The availability of these compounds can lead to local adaptation of GS pathway genes (Kliebenstein et al., 2001b). GS are derived from amino acids and can accordingly be divided into three main groups: indolic, aromatic, and aliphatic. The molecular mechanisms of the GS biosynthesis pathway have been reviewed and described extensively elsewhere (e.g., Halkier and Gershenzon, 2006; Redovnikovic et al., 2008; Sønderby et al., 2010). The diversity of aliphatic GS is, among others, due to different chain lengths and side chain modifications. *BCAT3* and *GS-ELONG* genes regulate chain length (Magrath et al., 1994; Kliebenstein et al., 2001b; Knill et al., 2008) and *AOP1-3* and *FMO-GS-OX1-5* modify the side chains (Kliebenstein et al., 2001c; Li et al., 2008).

*Arabidopsis thaliana* is also an important model species for understanding the molecular mechanisms regulating flowering time (Bouché et al., 2016 and the references therein). The switch from the vegetative to reproductive phase is one of the most important transitions in the life-cycle of a plant, moderated by abiotic and biotic cues. A plant needs to defend its vegetative and its new valuable generative tissues against potentially different herbivore attackers. Hence, shifts between plant development and plant defense traits can be critical to plant fitness. Jensen et al. (2015) found a link between the GS pathway and flowering time. They incorporated the aliphatic side-chain modifiers, *GS-AOP* genes, in an *AOP-0* background and found that they changed the flowering time of *A. thaliana*. Whether shifts in GS profiles and life-history transitions are seen in other Brassicaceae could establish that this is a general principle of crucifer evolution.

**FIGURE 1:**
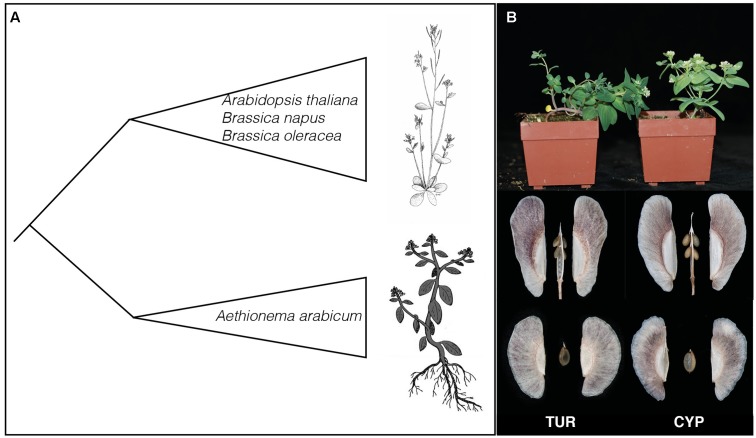
**Evolutionary position of *Aethionema arabicum* and parental lines used. (A)** Cartoon representation of the phylogenetic relationship between *Ae. arabicum* and the rest of the Brassicaceae. The species in the Brassicaceae core-group are examples. The drawings represent *Arabidopsis thaliana* (drawn by Mariet de Geus) and *Ae. arabicum* (adapted from Lenser et al., 2016). **(B)** Photographs of the two lines TUR and CYP used here. Shown are the habitus of the two lines and their dehiscent and indehiscent fruits and seeds. Plants on the left are TUR and on the right CYP. Photographs are made by Petra Bulankova and Arshad Waheed.

The annual species *Aethionema arabicum* belongs to the sister lineage to the rest of the Brassicaceae family and hence is at an important position for genomic and genetic comparisons of trait evolution (Schranz et al., 2012; **Figure 1A**). For example, *Ae. arabicum* is being used to study several aspects of life-history evolution including the molecular mechanisms of fruit and seed heteromorphism (Lenser et al., 2016). *Ae. arabicum* grows on steep stony slopes mainly in Iran and Turkey, although populations have also been found in Cyprus and Bulgaria (Velchev, 2015). Populations of *Ae. arabicum* go through their entire life-cycle between April and June, just before the summer heat strikes (Bibalani, 2012). However, the flowering time varies throughout the species distribution. The completion of the *Ae. arabicum* genome has also made it possible to study and show the synteny of GS genes between *A. thaliana* and *Ae. arabicum* (Haudry et al., 2013; Hofberger et al., 2013). However, the diversity of GS profiles in *Ae. arabicum* are not yet described.

The focus of this study is twofold. First, we describe the GS content of different tissues at different developmental stages for *Ae. arabicum*. We used two different *Ae. arabicum* accessions (TUR and CYP) differing in their life histories. We found that GS content in the leaves depends on the plants developmental stage. Moreover, we found that GS side-chain modifications are tissue specific and that the two *Ae. arabicum* accessions had a 10-fold difference of GS concentration in the fruits and a twofold difference in the leaves. Second, we identified the genomic locations controlling the GS profiles in *Ae. arabicum* by multi-trait and multi-environment quantitative trait locus (QTL) analyses using RIL populations developed from TUR and CYP. In total, we found five QTLs including one major QTL. Two QTL intervals contain homologs of *BCAT3* and *MYB28*, involved in the regulation of long-chained GS formation (Sønderby et al., 2010). Although none of the GS pathway homologs were located under our major QTL, this interval contains homologs of the sulfate transporter *SULTR2;1* and the flowering time regulator *FLOWERING LOCUS C (FLC)*. We argue that *Ae. arabicum* is a valuable system to understand the link between development and defense and that our study is an important first step in that direction.

## Materials and Methods

### Plant Material

Glucosinolates profiles and quantities of *Ae. arabicum* during development were measured in seeds, leaves, flowers, and fruits of two *Ae. arabicum* lines CYP and TUR. Seeds from the CYP individual originate from the pillow lavas of Kato-Moni in Cyprus (lat 35.057310 and lon 33.091832). The TUR individual is from the living plant collection of the Botanical Garden in Jena, Germany. However, population structure analyses have shown that this line originates from Turkey (Mohammadin et al., unpublished data). CYP and TUR have different life histories and growth characteristics. CYP has a more erect habit than TUR (**Figure 1B**) and CYP flowers very fast with only four leaves before flowering, while the TUR accession flowers from nine leaves onward. The CYP and TUR genotypes were used as parents to establish an F8 recombinant inbred line (RIL) population. For the QTL analyses we measured GS content in seeds (110 RILs), infructescences and leaves (99 RILs), all from the same mapping population.

For the GS through development as well as QTL experiments, seeds were germinated by placing them on a wet filter paper (demi-water) in a Petri dish sealed with Parafilm. However, the germination procedure differed between the two experiments. To measure GS content through development CYP and TUR seeds were imbibed at 18°C. Seeds showing a radicle after imbibition were sown directly in 12 cm pots, with five in each pot. Seeds for the QTL experiments were stratified at ∼4°C after which they were incubated at 18°C to allow germination. Seedlings for the QTL experiments were individually sown in 10 cm pots. Both experiments were conducted in the climate controlled greenhouse, at Wageningen UR with long day conditions (16 h light: 8 h dark) at 20°C.

To measure GSs during development, parental plants (CYP and TUR) were sampled weekly from the start of the experiment up to 8 weeks. *Ae. arabicum* does not have a rosette; hence cauline leaves of various ages were sampled and pooled to obtain the GS content throughout the plant. Seedlings (cotyledons and roots), leaves, flowers, immature fruits, and mature fruits where sampled separately and in triplicate (three different plants of the same line). Sampling was always done at 11:00 AM, taking diurnal GS variation into account (Petersen et al., 2002).

For the QTL experiment, leaves were collected when the plants had six fully developed leaves. All plants then showed reproductive buds or were fully flowering. Per line, leaves from two individuals were pooled. Reproductive tissues (combining infructenscence, flowers, and fruits; this is later referred to as ‘fruits’) were collected from the main stem of every RIL and parents 1 month after the start of the experiment. All freshly collected samples were immediately stored in liquid nitrogen and stored at -80°C. To assess the GS QTL(s) in seeds, we used dry ripened seeds harvested in 2014 from 110 RILs.

Frozen samples were freeze-dried at -40°C for 24 h. For GS extraction, samples were ground with 3 mm glass beads to obtain 2–10 mg of material. For the GS measurements through development: if there was <5 mg of material, samples were pooled from the same tissue of one parent before GS extraction. To analyze the QTL for seed GS ∼10 mg of seeds from all RILS were used for GS extraction.

### GS Extraction and Measurements

Leaves were freeze-dried until constant weight and ground to a fine powder. Between 2 and 10 mg of freeze dried leaves or 10 mg of seeds were extracted with 1 mL of 80% methanol (v:v) containing 0.05 mM intact 4-hydroxybenzylglucosinolate as internal standard (analysis of *Ae. arabicum* samples without addition of internal standard had shown that the samples do not contain 4-hydroxybenzylglucosinolate). After centrifugation, extracts were loaded onto DEAE Sephadex A 25 columns. Columns were washed with 1 ml 80% (v:v) methanol, 1 ml water, and 1 ml 0.02 M MES buffer (pH 5.2), before 50 μl of sulfatase solution (arylsulfatase from Sigma-Aldrich) was added. After incubation at room temperature overnight, desulfated glucosinolates were eluted with 0.5 mL water. The eluted desulfoglucosinolates were separated using high performance liquid chromatography (Agilent 1100 HPLC system, Agilent Technologies) on a reversed phase C-18 column (Nucleodur Sphinx RP, 250 mm × 4.6 mm, 5 μm, Machrey-Nagel, Düren, Germany) with a water (A)-acetonitrile (B) gradient (0–8 min, 10–50% B; 8–8.1 min, 50–100% B; 8.1–10 min 100% B and 10.1–13.5 min 10% B; flow 1.0 mL min^-1^). Detection was performed with a photodiode array detector and peaks were integrated at 229 nm. We used the following response factors: aliphatic glucosinolates 2.0, indole glucosinolates 0.5 (Burow et al., 2006) for quantification of individual glucosinolates. For identification of GS, some desulfoglucosinolate extracts were run on an LC-ESI-IonTrap-MS-system (Bruker Esquire6000) in positive ionization mode and compared to known GS from *A. thaliana* ecotype Col-0 leaf and seed extracts and for 3MSOOP to an *Erysimum cheiri* (type: Borntal-Lichter; Chrestensen) seed extract. Obtained mass spectral data of desulfoglucosinolates were compared to data shown in Kusznierewicza et al. (2013).

### Statistical Analyses

Statistical analyses of the development through time was not possible, because of limited sample sizes for all tissues at every time point (*n* = 3 or less due to pooling). Despite the lack of sample size, we found interesting patterns of GS change through time.

The linkage map used here contains 11 linkage groups and is based on 746 SNP from genotype by sequencing analysis of 167 RILs with the *Ae. arabicum* v2.5 used a reference genome (Nguyen et al., in preparation).

Genome-wide QTL analyses were done in Genstat (Payne et al., 2009) with a step-size of 5 cM. Single-trait, multi-trait and multi-environment QTL analyses were done for every tissue separately as done by Wei et al. (2014). This pipeline includes a single interval mapping (SIM) followed by a composite interval mapping (CIM) and a final model selection step for the single trait as well as multi-trait analyses. While single-trait QTL analyses infer QTLs per trait, multi-trait QTL analyses take all the traits simultaneously into account. This makes it possible to assess whether a QTL has a significant effect on a trait. A multi-trait analyses can infer the effect and location of the QTL for every trait using a backward selection of the found QTLs (Wei et al., 2014 and the references therein). A multi-environment linkage analysis works in a similar way as a multi-trait analysis, but now the effect of the environment (here the different tissues) on the QTLs per compound is assessed. R/qtl (Broman et al., 2003) was used to asses an interval of 1.5 LOD expanded to the markers to assess the genes underlying the QTL. R/qtl makes it possible to include bootstrapping to get the 1.5LOD confidence interval. The significant QTLs were named according to their linkage group, followed by a number depending on the QTL location (e.g., Q1.2 would be the second QTL found on linkage group 1).

Hofberger et al. (2013) assessed the homology of all GS pathway genes between *A. thaliana* and the *Ae. arabicum* v1 genome. We used SynFind (Lyons and Freeling, 2008) and the *Ae. arabicum* v2.5 genome in CoGe (Nguyen et al., in preparation; Lyons and Freeling, 2008) to establish if any of the homologs found by Hofberger et al. (2013) of the GS pathway occur between a one marker-interval from our QTLs. We used WUBlast from the Arabidopsis information resource (Huala et al., 2001) (with a significance cut-off e-value ≥ e^-10^, including introns and UTRs). Hence, we could confirm the location of homologs between *Ae. arabicum* and *A. thaliana* for our major QTL. Moreover, we used the transcriptomes of CYP and TUR from Mohammadin et al. (submitted) to assess whether the genes under major QTLs were expressed and contained single nucleotide polymorphisms (SNPs). These are transcriptomes from pooled tissues and developmental stages, varying from seed to leaves, and from seedlings to adult plants. SNP quality cut-off value was set as GQ ≥ 40 from the variant calls.

## Results

### Glucosinolates during Plant Development in *Aethionema arabicum*

To investigate if GS contents change through *Ae. arabicum* plant development (temporal) and to assess the GS composition in different tissues (spatial) we measured the GS compounds of *Ae. arabicum* for the two parental lines CYP and TUR from seed to a fully generative stage.

**FIGURE 2:**
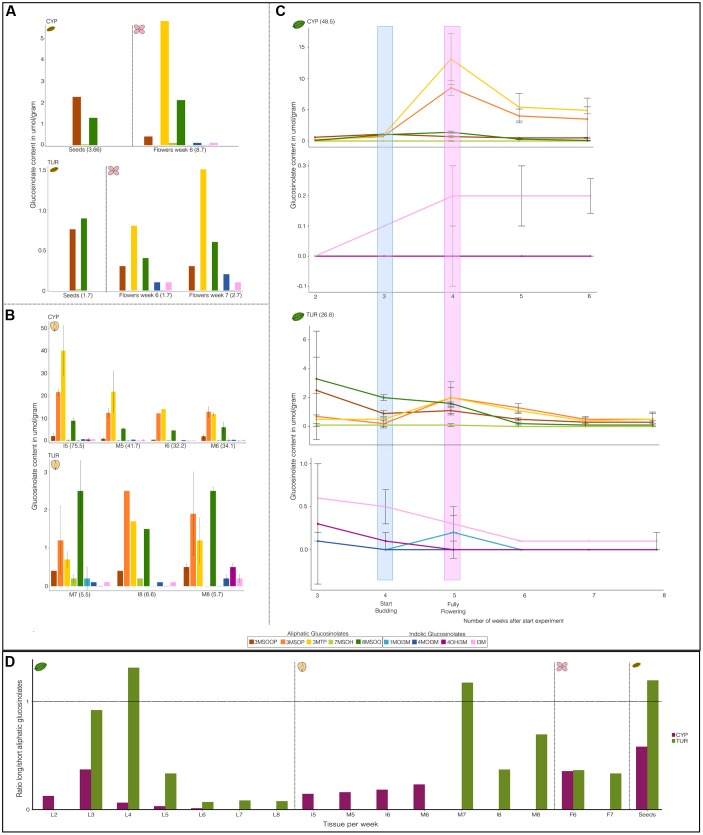
**Time course of glucosinolate (GS) composition of different tissues of *Aethionema arabicum* accessions CYP and TUR.** Shown are the averages and standard deviations. Bars and points with standard deviations have a sample size of *n* = 3. Bars and points without standard deviations were from pooled tissue (hence *n* = 1). All y-axes have a different scale. Numbers in brackets are total GS content in umol/gram dry weight. **(A)** Seed and flower GS. **(B)** Fruit GS. I = immature fruit, M = Mature fruit. Numbers of the abbreviations along the x-axis are the weeks from the start of the experiment. **(C)** Leaf GS: for every line (CYP or TUR) the top graph are the aliphatic GS and the bottom the indolic GS. Highlighted are the number of weeks after which plants start budding (blue) or are in full bloom (pink). **(D)** Ratio of long versus short aliphatic glucosinolates (GS). The ratio is calculated as the sum of all C7 and C8 GS divided by all C3 GS. The x-axis shows the tissues (L = leaves, I = immature fruits, M = mature fruits, F = flower, Seeds = ripened seeds). Numbers following the abbreviations are the weeks after the start of the experiment. Vertical dotted lines divide the graph into the different tissues.

There were a total of nine different GS compounds detected in *Ae. arabicum* (**Figure 2**). *Ae. arabicum* seeds contain only three GS that are all aliphatic and derived from the amino acid methionine: 3MSOOP (3-methylsulfonylpropyl, C_11_H_21_NO_11_S_3_), 7MSOH (7-methylsulfinylheptyl, C_15_H_29_NO_10_ S_3_), and 8MSOO (8-methylsulfinyloctyl C_16_H_31_NO_10_S_3_) GS (**Figure 2**). 8MSOO was the only compound that occurred in all tissues (flowers, fruits, and leaves). In addition to 8MSOO *Ae. arabicum* leaves and fruits also contained the Met-derived 3MSOP (3-methylsulfinylpropyl, C_11_H_21_NO_10_S_3_), 3MTP (3-methylthiopropyl, C_11_H_21_NO_9_S_3_) GS and the indolic tryptophane-derived I3M (indolyl-3-methyl, C_16_H_20_N_2_O_9_S_2_), 4MOI3M (4-hydroxy-indolyl-3-methyl, C_17_H_22_N_2_O_10_S_2_), 4OHI3M (4-methoxy-indolyl-3-methyl, C_17_H_21_N_2_O_10_S_2_), and 1MOI3M (1-methoxy-indolyl-3-methyl, C_17_H_22_N_2_O_10_S_2_) GS (**Figure 2**). Thus, compounds differ both in chain-length elongation and in side-chain modifications with a different number of oxygen and sulfur atoms creating sulfinylalkyls, sulfonylalkyls, and thioalkyls for aliphatic GS and adding methoxy- groups to the indolic GS. The greatest variety of compounds was found in the early developing leaves and fruits of TUR (**Figures 2B,C**), although this variation decreased through time in the leaves.

Differences between the ratio of different GS compounds can be a valuable method to examine differences between genotypes (Schranz et al., 2009). The GS type and quantity changed through time and varied between tissues (**Figure 2** and **Supplementary Table S1**). In most tissues the ratio of long- vs. short-chained aliphatic GS was skewed toward the short-chain compounds (**Figure 2D**). For CYP as well as for TUR GS content decreases after fruiting begins (**Figure 2C** and Supplementary Figure S1). CYP shows an increase of aliphatic GS after budding that peaks during flowering (**Figure 2C** and Supplementary Figure S1). It is not clear whether GS also increase in TUR after budding, as we do not have any data for the early seedlings of TUR (week 2). CYP has a higher GS content than TUR in most tissues. The only exception to this are the leaf indolic GS, where TUR starts on average with a higher indolic GS content than CYP (**Figure 1C**; CYP = 0 μmol/gram for all indolic GS and TUR 1MOI3M = 0.1 μmol/gram; 4MOI3M = 0.1 μmol/gram; 4OHI3M = 0.3 μmol/gram; I3M = 0.6 μmol/gram). Although the TUR indolic GS decrease toward 0.1 μmol/gram or even less, the CYP indolic I3M increases through time.

### QTL Analyses

To understand the genetic regulation of GS in *Ae. arabicum* we investigated the GS profiles and identified the genomic locations underlying the GS phenotype in *Ae. arabicum* leaves, fruits and seeds from RILs and their parental lines TUR and CYP. In addition to single-trait QTL analyses we also applied multi-trait and multi-environment QTL analyses to assess the effects of the QTLs on the different compounds and on the different tissues.

The leaf and fruit samples of the RILs were taken after budding or even during flowering and contain only 3MSOP, 3MTP, 8MSOO, 4OHI3M, and I3M. The segregation spectrum of the RILs was similar for most GS whether they were isolated from leaves, fruits or seeds (Supplementary Figure S2). However, the GS concentrations depended on the particular compound and tissue, with indolic GS being lower than aliphatic GS (Supplementary Figure S2). While the distribution of the GS concentrations of RIL lines were more mostly intermediate between the parental values, in particular for seed GS concentrations differed for some compounds between RIL and parental lines (Supplementary Figure S2).

For the single-trait single-environment analysis, we found six different QTLs on four different linkage groups Q5.1 on LG5, Q6.1 and Q6.2 on LG6, Q8.1 and Q8.2 on LG8 and Q10.1 on LG10 (**Table 1**). Three of the QTLs (Q6.1, Q6.2, and Q8.2) also occur in the multi-trait and multi-environment QTL analyses (**Table 2** and **Supplementary Table S2**). Q8.2 is a major QTL throughout all our analyses. The single-trait single-environment analysis shows a QTL for the indolic 4OHI3M. However, this peak is not present in the multi-environment QTL analysis (**Supplementary Table S2**).

**Table 1:** Significant QTLs from single trait analyses in *Aethionema arabicum* TURxCYP recombinant inbred lines.

Tissue	GS^a^	QTL^b^	Marker	Position (cM)^c^	Lower–Upper^d^	-Log10(p)	AE^e^	SE^f^	PVE (%)^g^
Leaf	3MSOP	Q8.2	S44_973817	151.0	131.92–167.72	4.57	0.55	0.12	16.41
Leaf	3MTP	Q8.2	S44_827783	153.9	143.57–164.25	7.99	1.99	0.317	28.34^∗^
Leaf	8MSOO	Q6.2	S40_550522	78.8	61.78–95.83	6.26	0.40	0.075	19.44
Leaf	8MSOO	Q8.2	S44_609479	158.1	121.15–167.72	4.36	0.32	0.075	12.44
Leaf	A/I	Q10.1	S61_1993061	124.5	85.19–160.54	3.54	5.73	1.52	12.12
Leaf	A/I	Q8.2	S44_827783	153.9	123.08–167.72	4.28	6.06	1.43	13.55
Leaf	All GS	Q8.2	S44_609479	158.1	147.14–167.72	7.5	2.95	0.49	27.13^∗^
Fruit	3MSOP	Q8.2	S44_973817	151.0	140.99–160.92	8.26	2.98	0.46	30.74^∗^
Fruit	3MTP	Q6.2	S58_8426	72.8	30.92–114.76	4.48	1.78	0.41	12.43
Fruit	3MTP	Q8.2	S44_827783	153.9	139.37–167.72	7.3	2.42	0.41	22.92
Fruit	8MSOO	Q8.2	S44_827783	153.9	145.97–161.85	10.36	3.34	0.448	37.12^∗^
Fruit	4OHI3M	Q5.1	S53_4972590	25.0	0–71.94	4.07	0.42	0.10	11.94
Fruit	4OHI3M	Q8.2	S44_827783	153.9	141.47–166.35	7.62	0.61	0.10	25.78^∗^
Fruit	All GS	Q6.2	S58_8426	72.8	0–129.44	4.64	4.4	0.99	10.28
Fruit	All GS	Q8.2	S44_827783	153.9	145.39–162.43	11.68	8.12	0.99	34.99^∗^
Seed	3MSOOP	Q8.1	S81_435439	7.9	0.73–58.6	7.89	3.74	0.871	10.53
Seed	3MSOOP	Q8.2	S44_827783	153.9	143.48–164.34	9.34	5.96	0.87	26.81^∗^
Seed	7MSOH	Q6.1	S5_745413	16.6	0.0–41.18	5.62	0.15	0.03	14.6
Seed	7MSOH	Q8.2	S44_973817	151	139.57–162.35	8.81	0.2	0.03	24.99^∗^
Seed	8MSOO	Q8.2	S44_973817	151	140.04–161.88	7.57	6.37	1.06	25.84^∗^
Seed	All GS	Q8.2	S44_973817	153.9	146–161.83	10.17	33.7	1.79	13.03

*^a^Glucosinolate compound; A/I, ratio Aliphatic/Indolic; ^b^QTL; the first number corresponds to the linkage group; ^c^position along linkage group in centimorgan; ^d^lower and upper bound op QTL from Genstat; ^e^additive effect. Negative effects are from CYP, positive from TUR, Negative or positive effect means that the CYP or TUR allele has a stronger effect. ^f^Standard error of additive effect; ^g^percentage of explained variance. ^∗^Major QTL (PVE ≥ 25%, after Burke et al., 2002).*

**Table 2:** Significant QTLs from multi trait analyses in *Aethionema arabicum* TURxCYP recombinant inbred lines.

QTL	Marker	Tissue	LG^a^	Position (cM)^b^	-Log(p)	GS^c^	PVE %^d^
Q6.1	S5_745413	Leaf	6	16.57	4.34	3MTP 8MSOO	3.6 5.9
Q6.2	S40_63849	Leaf	6	74.62	5.66	3MTP 8MSOO I3M	4.0 15.5 6.9
Q8.2	S44_827783	Leaf	8	153.91	9.25	3MSOP 3MTP 8MSOO I3M	14.9 24.3 7.4 3.8
Q6.2	S40_63849	Fruit	6	74.62	5.05	3MSOP 3MTP 8MSOO 4OHI3M	6.4 12.9 3.6 4.3
Q8.2	S44_827783	Fruit	8	153.91	17.26	3MSOP 3MTP 8MSOO 4OHI3M	24.0 24.4 34.1^∗^ 26.8^∗^
Q6.1	S5_745413	Seed	6	16.57	9.28	7MSOH 8MSOO	16.8 6.6
Q2.1	S13_476613	Seed	2	167.30	7.56	3MSOOP 7MSOH	3.0 3.5
Q8.1	S93_383588	Seed	8	11.54	4.14	3MSOOP	7.2
Q8.2	S44_827783	Seed	8	153.91	10.54	3MSOOP 7MSOH 8MSOO	26.6^∗^ 25.7^∗^ 18.5

*The percentage of variability explained is shown for every GS compound that had a significant (α ≤ 0.05) interaction with the QTL.*

*^a^Linkage group; ^b^marker position in centimorgans; ^c^significant glucosinolate compounds (α ≥ 0.05); ^d^percentage of variance explained. ^∗^Major QTL (PVE ≥ 25%, after Burke et al., 2002).*

**FIGURE 3:**
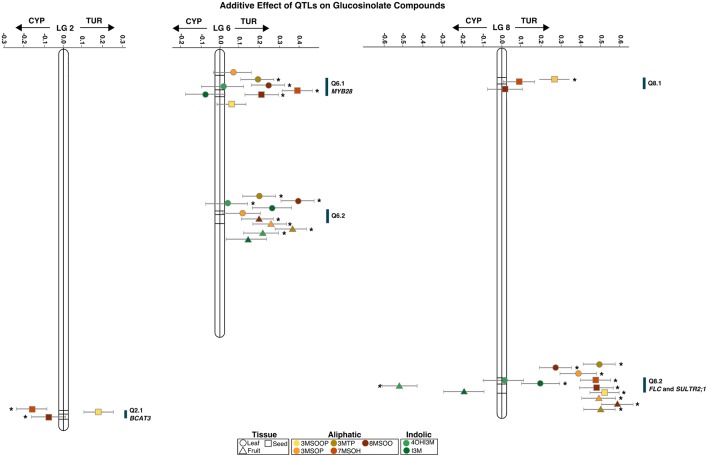
**Additive effect of QTLs from a multi-trait QTL analysis for different glucosinolates (GS) from different tissues of *Aethionema arabicum*.** Shown are the Linkage Groups (numbers above the x-axis) with lines in them for the QTL. The middle line is always the main locus; the other two are its closest markers. Positive effects are from the TUR allele, and negative values from the CYP allele. Points represent the tissues (different shapes) and GS compounds (different colors, see legend at bottom of figure) with their standard error (gray whiskers). QTL with a significant (*P* < 0.05) effect on the GS are denoted with an asterisk. Blue bars along the linkage groups represent the QTL position and candidate genes. All points belonging to the same QTL are off-set for visibility.

The multi-trait single environment analysis shows that 4OHI3M is only significantly affected by the fruit QTLs (**Figure 3**). The indolic I3M significantly contributes to Q8.2 in the leaves (**Figure 3**). The multi-trait QTL (**Figure 3**) shows that aliphatic GS in all tissues significantly contribute to Q8.2. There is a difference in contribution between 3MSOP in leaves and fruits: while 3MSOP has a significant contribution with both QTLs in fruits there is only the significant contribution with Q8.2 in leaves (**Figure 3**). 3MTP however has a significant contribution with all the QTLs in leaves. Hence, the occurrence of 3MTP or 3MSOP seems to be tissue specific. Moreover, compared to leaves and fruits, seeds have two unique loci: Q2.1 and Q8.1.

The multi-trait (**Figure 3**) and multi-environment (**Supplementary Table S2**) analyses both show a large effect from the TUR allele for Q6.2 and Q8.2 in leaves and seeds, and for Q6.2 in leaves and fruits. However, Q2.1 in seeds (for 7MSOH and 8MSOO) had a large effect from the CYP allele. Q8.2 had a large effect from the CYP allele for the indolic GS, which was also the case for leaf Q6.1 (**Figure 3**). The combination of the low GS levels for TUR compared to CYP and the large effect of the TUR allele on the QTLs suggests that these QTLs could be inhibitors of GS synthesis.

As leaves and fruits contain the same compounds, we used a multi-environment QTL analyses to assess the effect of the QTLs on the tissues for every compound separately. In addition to the already shown QTLs of the single trait single environment analysis and the multi-trait single environment analysis, the multi-environment single trait analysis identified three new QTLs: Q1.1, Q3.1, and Q8.3 (**Supplementary Table S2**). This comparison shows that the QTLs are significantly correlated mainly in fruits. An exception to this is 3MTP where both leaves and fruits are significantly correlated with the QTLs.

Using the homology and synteny between *A. thaliana* and *Ae. arabicum* of GS pathway genes (Hofberger et al., 2013) we assessed whether any of the genes were potential candidate genes for any of our QTLs. We found *BCAT3* was within the confidence interval of Q2.1 and *MYB28* was in the confidence interval of Q6.1. None of the GS pathway genes were coded by the *Ae. arabicum* genes under the major QTL, Q8.2, that appears in every comparison. Using WU-BLAST (Huala et al., 2001; **Supplementary Table S3**) we identified a total of 87 genes within the confidence interval. Using the raw genotype information from Nguyen et al. (in preparation) we were able to define more precisely the region to 58 kB and 15 candidate genes (**Supplementary Table S3**). The genes have diverse functions including fatty acid biosynthesis, ethylene-activated signaling, proteolysis, Pollen Ole e1, one unknown protein, the sulfate transporter *SULTR2;1* and *FLOWERING TIME LOCUS C*.

Eight out of the 15 genes within the 58 kB interval had SNPs within the transcriptomes (mRNAs) of Mohammadin et al. (submitted; **Supplementary Table S3**). Although *SULTR2;1* has SNPs in this transcriptome dataset, we only identified a single SNP in *FLC* according to our cut-off values (there was one SNP in *FLC* with GQ = 39, our cut-off was GQ ≥ 40). However, the QTL effect could instead be caused by upstream regulatory differences.

## Discussion

Here, we present the correlation between the reproductive phase change and the composition of defense compounds in the annual Brassicaceae *Ae. arabicum*. Although the GS pathway has been extensively studied in *A. thaliana*, the information from a phylogenetically distant crucifer may elucidate alternative regulators of the glucosinolate pathway. We show that the major genomic location (Q8.2) associating with GS variation contains 15 genes, among which are the sulfur transporter *SULTR2;1* and the *FLOWERING TIME LOCUS C* (*FLC*), genes that are not yet reported to be directly involved in the GS biosynthesis pathway. Interestingly, the faster flowering ecotype (CYP) also has the higher constitutive glucosinolate content, which is contrast to the prevailing model of defense and fitness allocation costs.

Glucosinolates type and quality changes throughout the development of two *Ae. arabicum* individuals. This is most clearly seen in CYP where between the onset of budding and full-bloom there is an increase in aliphatic GS (**Figure 2** and Supplementary Figure S1). Moreover both in CYP and TUR the level of GS decreases after flowering (**Figure 2** and Supplementary Figure S1). Combining the change in GS throughout *Ae. arabicum*’s development and the large difference of GS concentration found between CYP and TUR strongly suggests some regulatory factor(s) other than the known genes involved in the GS biosynthesis pathway. The multi-trait and multi-environment QTL analyses indeed show one major QTL (Q8.2) explaining up to 37% of the variation between the RILs (**Table 1**). Preliminary fine mapping this region indicated 15 genes, including the intriguing candidates the sulfate transporter *SULTR2*;*1* and *FLOWERING TIME LOCUS C* (*FLC*, one of the MADS-box transcription factors that regulated flowering in Brassicaceae (Ietswaart et al., 2012; Bouché et al., 2016).

The importance of sulfur for GS and its locality under our major QTL might indicate an indirect relation between sulfur transport and GS formation. *SULTR2;1* is involved in the root to shoot sulfate transport (Gigolashvili and Kopriva, 2014). Sulfur is an essential macronutrient for plant development (Gigolashvili and Kopriva, 2014). Sulfur is used in the biosynthesis of several compounds varying from amino acids to proteins, co-enzymes, vitamins and defense metabolites like GS (Falk et al., 2007; Gigolashvili and Kopriva, 2014). With at least two sulfur atoms GS can include ∼30% of the plants sulfur (Aarabi et al., 2016). The addition of sulfur can increase GS levels with 25–50%, depending on the amount of sulfur and the treatment (Falk et al., 2007). There are four groups of sulfate transporters: high affinity transporters (*SULTR1*’s); plastid membrane transporters and low affinity transporters such as (*SULTR2’*s); transporters of the symbiosome membrane of the legume:rhizobia symbiosis (*SULTR3*’s); and transporters with an unknown function (*SULTR4*’s) (Gigolashvili and Kopriva, 2014). The low affinity sulfate transporters, such as *SULTR2;1* depend more on sulfur availability and hence respond quicker to sulfur deficiency (Falk et al., 2007). Under sulfur deficient circumstances GS are broken down and used as a sulfur source (Falk et al., 2007) while the biosynthesis of GS is repressed (Aarabi et al., 2016). The natural growing area of *Ae. arabicum* are mainly steep stony slopes. There is little organic matter in the soil and thus sulfur concentrations might be limiting and what would be available would be susceptible to loss due to leaching. Growth of plants under potential low sulfur availability and the regulation of sulfur transport and use (and impact on GS levels) needs to be further investigated in *Aethionema*.

Plant defense and the transition from a vegetative to a generative life stage can have an effect on one another. For example, the biosynthesis of GS can reduce fitness in *A.* *thaliana* (Kliebenstein, 2004). In *A. thaliana* and *Brassica napus* the production of a new leaf leads to an increase of GS concentration in the new leaf compared to the older leaves (Brown et al., 2003). Many tissues of Brassicaceae species express the MADS-Box gene *FLOWERING TIME LOCUS C* (*FLC)* throughout their life-cycle. However, *FLC* is primarily known as a floral repressor in meristems where expression is stably repressed upon prolonged cold-treatment or vernalization (Deng et al., 2011; Ietswaart et al., 2012; Bouché et al., 2016). *Ae.* *arabicum* is a relatively fast flowering annual and does not require vernalization. Our data show that the levels of leaf GS increase between the onset of budding and full-bloom in *Ae. arabicum*. This implies that the *Ae. arabicum FLC* is involved in the regulation of GS biosynthesis. *FLC* has more than 500 binding sites in the *A. thaliana* genome with *CYP79B3* being one of them (Deng et al., 2011). *CYP79B3* belongs to the cytochrome P450 *CYP79* family and is involved in the formation of the core structure of indolic GS (Sønderby et al., 2010). In addition, compared to wild type plants *MYB29* is down-regulated in *FLC* knock outs while *MYB51* and *SOT16* are up-regulated (Mateos et al., 2015). *MYB28* and *MYB29* are essential for the biosynthesis of aliphatic GS (Hirai et al., 2007). *MYB51* controls the formation of indolic compounds together with *MYB34* and *MYB122* (Frerigmann and Gigolashvili, 2014) and it has been shown that *SOT16* catalyzes the final step of indolic GS formation (Piotrowski et al., 2004). The differential expression of essential GS pathway genes in *FLC* knock-outs (Mateos et al., 2015) shows a cross-talk between GS biosynthesis and flowering time. The major QTL (Q8.2) found here in *Ae. arabicum* also indicates a link between GS biosynthesis and development. Jensen et al. (2015) showed that the introduction of the *GS*-*AOP* genes in *AOP-0* lines does not change the GS levels, but influences flowering time. This effect depended on the genetic background and could vary between an increase and decrease of flowering time (Jensen et al., 2015). They hypothesized that *AOP2* and *AOP3* could mediate the cross talk between flowering and defense. Our major QTL (Q8.2) shows a likely relationship between flowering time and defense. This relationship could depend on a cross-talk between both pathways. Differences in epigenetic marks and/or gene-regulatory elements could explain differential expression of CYP and TUR alleles at *FLC* and is currently being investigated.

*Aethionema arabicum* ripened seeds, from CYP as well as from TUR, have lower GS diversity than *Ae. arabicum* fresh leaves. This differs from other crucifers, e.g., *A. thaliana, Brassica oleracea* and *B. napus*, where the GS diversity and concentration are the highest in the seeds and decrease in the following order in the inflorescence, siliques, leaves and roots (Brown et al., 2003; Velasco et al., 2008; Sotelo et al., 2014). *Ae. arabicum* fresh fruits, including seeds, have very high GS levels (**Figure 2**) comparable to the levels found in *A. thaliana* seeds (Brown et al., 2003). While all *Ae. arabicum* tissues contain indolic GS, their ripened seeds lack these compounds. The difference in indolic GS is also seen between the seeds and leaves of *A. thaliana, B. oleracea*, and *B. napus* (Kliebenstein et al., 2001a; Petersen et al., 2002; Brown et al., 2003; Velasco et al., 2008; Sotelo et al., 2014). Aliphatic GS are known to have a negative effect on the survival and growth of herbivorous insects (Beekweelder et al., 2008) explaining the persistence of aliphatic GS in the 2-year-old *Ae. arabicum* seeds, but also the presence of GS in seeds of Brassicaceae that lack GS in their vegetative tissue (Windsor et al., 2005). The difference between young versus old tissue might indicate a breakdown process of (indolic) GS in the seed cells over time or a translocation process (away from the seeds) as the seeds ripen, though it might also be correlated to the developmental stage of ripened seeds, hypotheses still to be tested.

The two parental ecotypes used here (CYP and TUR) had a 10-fold difference in GS concentrations in the fruits and twofold difference concentration in leaves, with the earlier-flowering CYP genotype always having the higher aliphatic GS concentration (**Figure 2**). These differences seem to reflect the extremes found in other Brassicaceae species. For example Italian horseradish (*Armoracia rusticana*) roots can differ up to twenty times in GS concentration depending on the accession (Agneta et al., 2014); American wild radish (*Raphanus raphanistrum*) accessions has more than 20x difference in GS concentrations in its secondary branches of plants from North Carolina vs. Mississippi (Malik et al., 2010); and *A. thaliana* leaves can have extremes of more than 10x differences in total GS concentrations (Kliebenstein et al., 2001b). In *Boechera stricta*, there are also differences in both the total content (quantitative) and type (qualitative) of GS between genotypes (Schranz et al., 2009; Manzaneda et al., 2010). However, in *B. stricta* the late-flowering ecotypes generally have the higher total content (Manzaneda et al., 2010).

The different tissues of *Ae. arabicum* all show a skewed ratio of long- versus short-chain GS and different side chain modifications. A similar pattern has been shown in *A. thaliana*, where ecotypes with higher amounts of C3 GS had lower C8 to C7 ratios (Kliebenstein et al., 2001b). The negative long-short correlation could be a biochemical effect whereby short GS precursors are kept in the chain elongation loop as long as they are not used (Olson-Manning et al., 2015). *Ae. arabicum* has tissue specific oxidization levels of GS side chain modifications: leaves were mainly correlated with 3MTP, while 3MSOP is linked to fruits and 3MSOOP occurs only in the flowers and seeds (**Figure 2**), this is also reflected in the multi-trait QTLs (**Figure 3**). Side chain oxidation (OHP vs. 3MSOP) has a negative effect on the weight gain of herbivores (Rohr et al., 2009) presenting an array of testable hypotheses for the ecological effect of highly oxidized GS in *Ae. arabicum* seeds. Knowing the genetic architecture controlling GS variation in *Ae. arabicum* can elucidate GS regulation and tissue specific side chain modifications. Moreover the correlation between the long and short aliphatic GS indicates a similar regulatory factor. However, none of the expected GS pathway genes, e.g., *MAM’*s, *CYP’s, GS-OX*’s, or *AOP’s* were associated with identified under our QTLs. Only two of the minor QTLs contain genes involved in the chain elongation: *BCAT3* and *MYB28* (Beekweelder et al., 2008; Knill et al., 2008). *BCAT3* is involved in the chain elongation process and *BCAT3* knockouts increase the level of long chain GS compounds (Knill et al., 2008; Sønderby et al., 2010). Although, it is not known how *MYB28* is involved in the long-chain GS biosynthesis it has been shown that the knockout *myb28* blocks the expression of long-chain GS (Beekweelder et al., 2008).

Our *Ae. arabicum* lines show that a during the transition from vegetative tissue to generative tissue correlates with a transition in GS content. Also, we consistently find that the early flowering CYP genotype has a higher constitutive GS content than the later flowering TUR genotype. QTL analyses for GS content of three different tissues all point to one major QTL containing two potential candidates *SULTR2;1* and *FLC*. Future gene expression analysis, transformation experiments, fine mapping and phenotyping of more accessions could help to understand whether and if so in which way these genes and traits are involved in the plants defense pathway. Although the focus of our research has mainly been a genetic one, future research should also focus on the effect of the GS biosynthesis pathway on herbivores and herbivory.

## Author Contributions

SM conducted the GS through development experiment, did all the analyses and wrote the paper. T-PN revised the manuscript and helped with the QTL analyses. MvW conducted the QTL experiments. MR performed the GS analyses and revised the manuscript. MS led the experiments and revised the paper and figures.

## Conflict of Interest Statement

The authors declare that the research was conducted in the absence of any commercial or financial relationships that could be construed as a potential conflict of interest.

## Supplementary Material

TABLE S1 **Average glucosinolate values of *Aethionema arabicum* lines TUR and CYP leaves and their standard deviations.** The numbers after the tissues are number of weeks after start of experiment.

TABLE S2 **Significant QTLs from multi environment analyses in *Aethionema arabicum* TURxCYP recombinant inbred lines**.

TABLE S3 ***Arabidopsis thaliana* homologs of *Aethionema arabicum* genes in the interval of the major Q8.2 and the fine-mapping details**.

## References

[B1] Aarabi F. Kusajima M. Tohge T. Konishi T. Gigolashvili T. Takamune M. (2016). Sulfur deficiency–induced repressor proteins optimize glucosinolate biosynthesis in plants. *Sci. Adv.* 2 1–18. 10.1126/sciadv.1601087 PMC5055385 27730214

[B2] Agneta R. Möllers C. De Maria S. Rivelli A. R. (2014). Evaluation of root yield traits and glucosinolate concentration of different *Armoracia rusticana* accessions in Basilicata region (southern Italy). *Sci. Hortic. (Amsterdam).* 170 249–255. 10.1016/j.scienta.2014.03.025

[B3] Beekweelder J. van Leeuwen W. van Dam N. M. Bertossi M. Grandi V. Mizzi L. (2008). The impact of the absence of aliphatic glucosinolates on insect herbivory in *Arabidopsis*. *PLoS ONE* 3:e2068 10.1371/journal.pone.0002068 PMC2323576 18446225

[B4] Bibalani G. H. (2012). Investigation on flowering phenology of *Brassicaceae* in the Shanjan region Shabestar district, NW Iran (usage for honeybees). *Ann. Biol. Res.* 6 1958–1968.

[B5] Bouché F. Woods D. Amasino R. M. (2016). Winter memory throughout the plant kingdom: different paths to flowering. *Plant Physiol.* 173 127–135. 10.1104/pp.16.01322 PMC5210730 27756819

[B6] Broman K. W. Wu H. Sen Churchill G. A. (2003). R/qtl: QTL mapping in experimental crosses. *Bioinformatics* 19 889–890. 10.1093/bioinformatics/btg112 12724300

[B7] Brown P. D. Tokuhisa J. G. Reichelt M. Gershenzon J. (2003). Variation of glucosinolate accumulation among different organs and developmental stages of *Arabidopsis thaliana*. *Phytochemistry* 62 471–481. 10.1016/S0031-9422(02)00549-6 12620360

[B8] Burke J. M. Tang S. Knapp S. J. Rieseberg L. H. (2002). Genetic analysis of sunflower domestication. *Genetics* 161 1257–1267. 12136028 10.1093/genetics/161.3.1257 PMC1462183

[B9] Burow M. Müller R. Gershenzon J. Wittstock U. (2006). Altered glucosinolate hydrolysis in genetically engineered *Arabidopsis thaliana* and its influence on the larval development of *Spodoptera littoralis*. *J. Chem. Ecol.* 32 2333–2349. 10.1007/s10886-006-9149-1 17061170

[B10] Deng W. Ying H. Helliwell C. A. Taylor J. M. Peacock W. J. Dennis E. S. (2011). FLOWERING LOCUS C (FLC) regulates development pathways throughout the life cycle of *Arabidopsis*. *Proc. Natl. Acad. Sci.* *U.S.A* 108 6680–6685. 10.1073/pnas.1103175108 21464308 PMC3081018

[B11] Edger P. P. Heidel-Fischer H. M. Bekaert M. Rota J. Glöckner G. Platts A. E. (2015). The butterfly plant arms-race escalated by gene and genome duplications. *Proc. Natl. Acad. Sci.* *U.S.A.* 112 8362–8366. 10.1073/pnas.1503926112 26100883 PMC4500235

[B12] Falk K. L. Tokuhisa J. G. Gershenzon J. (2007). The effect of sulfur nutrition on plant glucosinolate content: physiology and molecular mechanisms. *Plant Biol.* 9 573–581. 10.1055/s-2007-965431 17853357

[B13] Frerigmann H. Gigolashvili T. (2014). MYB34, MYB51, and MYB122 distinctly regulate indolic glucosinolate biosynthesis in *Arabidopsis thaliana*. *Mol. Plant* 7 814–828. 10.1093/mp/ssu004 24431192

[B14] Gigolashvili T. Kopriva S. (2014). Transporters in plant sulfur metabolism. *Front. Plant Sci.* 5:442 10.3389/fpls.2014.00442 PMC4158793 25250037

[B15] Halkier B. A. Gershenzon J. (2006). Biology and biochemistry of glucosinolates. *Annu. Rev. Plant Biol.* 57 303–333. 10.1146/annurev.arplant.57.032905.105228 16669764

[B16] Haudry A. Platts A. E. Vello E. Hoen D. R. Leclercq M. Williamson R. J. (2013). An atlas of over 90,000 conserved noncoding sequences provides insight into crucifer regulatory regions. *Nat. Genet.* 45 891–898. 10.1038/ng.2684 23817568

[B17] Hirai M. Y. Sugiyama K. Sawada Y. Tohge T. Obayashi T. Suzuki A. (2007). Omics-based identification of *Arabidopsis* Myb transcription factors regulating aliphatic glucosinolate biosynthesis. *Proc. Natl. Acad. Sci. U.S.A.* 104 6478–6483. 10.1073/pnas.0611629104 17420480 PMC1849962

[B18] Hofberger J. A. Lyons E. Edger P. P. Chris Pires J. Eric Schranz M. (2013). Whole genome and tandem duplicate retention facilitated glucosinolate pathway diversification in the mustard family. *Genome Biol. Evol.* 5 2155–2173. 10.1093/gbe/evt162 24171911 PMC3845643

[B19] Hopkins R. J. van Dam N. M. van Loon J. J. A. (2009). Role of glucosinolates in insect-plant relationships and multitrophic interactions. *Annu. Rev. Entomol.* 54 57–83. 10.1146/annurev.ento.54.110807.090623 18811249

[B20] Huala E. Dickerman A. W. Garcia-Hernandez M. Weems D. Reiser L. Lafond F. (2001). The *Arabidopsis* information resource (TAIR): a comprehensive database and web-based information retrieval, analysis, and visualization system for a model plant. *Nucleic Acids Res.* 29 102–105. 10.1093/nar/29.1.102 11125061 PMC29827

[B21] Ietswaart R. Wu Z. Dean C. (2012). Flowering time control: another window to the connection between antisense RNA and chromatin. *Trends Genet.* 28 445–453. 10.1016/j.tig.2012.06.002 22785023

[B22] Jensen L. M. Jepsen H. S. K. Halkier B. A. Kliebenstein D. J. Burow M. (2015). Natural variation in cross-talk between glucosinolates and onset of flowering in *Arabidopsis*. *Front. Plant Sci.* 6:697 10.3389/fpls.2015.00697 PMC4561820 26442014

[B23] Kliebenstein D. J. (2004). Secondary metabolites and plant/environment interactions: a view through *Arabidopsis thaliana* tinged glasses. *Plant Cell Environ.* 27 675–684. 10.1111/j.1365-3040.2004.01180.x

[B24] Kliebenstein D. J. Gershenzon J. Mitchell-Olds T. (2001a). Comparative quantitative trait loci mapping of aliphatic, indolic and benzylic glucosinolate production in *Arabidopsis thaliana* leaves and seeds. *Genetics* 159 359–370. 11560911 10.1093/genetics/159.1.359 PMC1461795

[B25] Kliebenstein D. J. Kroymann J. Brown P. Figuth A. Pedersen D. Gershenzon J. (2001b). Genetic control of natural variation in *Arabidopsis* glucosinolate accumulation. *Plant Physiol.* 126 811–825. 10.1104/pp.126.2.811 11402209 PMC111171

[B26] Kliebenstein D. J. Lambrix V. M. Reichelt M. Gershenzon J. Mitchell-Olds T. (2001c). Gene duplication in the diversification of secondary metabolism: tandem 2-oxoglutarate–dependent dioxygenases control glucosinolate biosynthesis in *Arabidopsis*. *Plant Cell* 13 681–693. 11251105 10.1105/tpc.13.3.681 PMC135509

[B27] Knill T. Schuster J. Reichelt M. Gershenzon J. Binder S. (2008). *Arabidopsis* branched-chain aminotransferase 3 functions in both amino acid and glucosinolate biosynthesis. *Plant Physiol.* 146 1028–1039. 10.1104/pp.107.111609 18162591 PMC2259058

[B28] Koroleva O. A. Davies A. Deeken R. Thorpe M. R. Tomos A. D. Hedrich R. (2000). Different myrosinase and ideoblast distribution in *Arabidopsis* and *Brassica* napus. *Plant Physiol.* 127 1750–1763. 10.1104/pp.010334 PMC133578 11743118

[B29] Kusznierewicza B. Iori R. Piekarska A. Namiesnik J. Bartoszek A. (2013). Convenient identification of desulfoglucosinolates on the basis of mass spectra obtained during liquid chromatography–diode array–electrospray ionization mass spectrometry analysis: method verification for sprouts of different Brassicaceae species extracts. *J. Chromatogr. A* 1278 108–115. 10.1016/j.chroma.2012.12.075 23352826

[B30] Lenser T. Graeber K. Cevik Ö. S. Adigüzel N. Dönmez A. A. Kettermann M. (2016). *Aethionema arabicum* as a model system for studying developmental control and plasticity of fruit and seed dimorphism. *Plant Physiol.* 172 1691–1707. 10.1104/pp.16.00838 27702842 PMC5100781

[B31] Li J. Hansen B. G. Ober J. A. Kliebenstein D. J. Halkier B. A. (2008). Subclade of flavin-monooxygenases involved in aliphatic glucosinolate biosynthesis. *Plant Physiol.* 148 1721–1733. 10.1104/pp.108.125757 18799661 PMC2577257

[B32] Lyons E. Freeling M. (2008). How to usefully compare homologous plant genes and chromosomes as DNA sequences. *Plant J.* 53 661–673. 10.1111/j.1365-313X.2007.03326.x 18269575

[B33] Magrath R. Bano F. Morgner M. Parkin I. Sharpe A. Lister C. (1994). Genetics of aliphatic glucosinolates. I. Side chain elongation in *Brassica napus* and *Arabidopsis thaliana*. *Heredity (Edinb).* 72 290–299. 10.1038/hdy.1994.39

[B34] Malik M. S. Riley M. B. Norsworthy J. K. Bridges W. (2010). Variation of glucosinolates in wild radish (*Raphanus raphanistrum*) accessions. *J. Agric. Food Chem.* 58 11626–11632. 10.1021/jf102809b 20964435

[B35] Mano Y. Nemoto K. (2012). The pathway of auxin biosynthesis in plants. *J. Exp. Bot.* 63 2853–2872. 10.1093/jxb/ers091 22447967

[B36] Manzaneda A. J. Prasad K. V. S. K. Mitchell-Olds T. (2010). Variation and fitness costs for tolerance to different types of herbivore damage in *Boechera stricta* genotypes with contrasting glucosinolate structures. *New Phytol.* 188 464–477. 10.1111/j.1469-8137.2010.03385.x 20663059 PMC2950872

[B37] Mateos J. L. Madrigal P. Tsuda K. Rawat V. Richter R. Romera-Branchat M. (2015). Combinatorial activities of SHORT VEGETATIVE PHASE and FLOWERING LOCUS C define distinct modes of flowering regulation in *Arabidopsis*. *Genome Biol.* 16:31 10.1186/s13059-015-0597-1 PMC4378019 25853185

[B38] Olson-Manning C. F. Strock C. F. Mitchell-Olds T. (2015). Flux control in a defense pathway in *Arabidopsis thaliana* is robust to environmental perturbations and controls variation in adaptive traits. *G* 3 5 2421–2427. 10.1534/g3.115.021816 PMC4632061 26362766

[B39] Payne R. W. Murray D. A. Harding S. A. Baird D. B. Soutar D. M. (2009). *GenStat for Windows*, 12th Edn Hemel Hempstead: VSN International.

[B40] Petersen B. L. Chen S. Hansen C. H. Olsen C. E. Halkier B. A. (2002). Composition and content of glucosinolates in developing *Arabidopsis thaliana*. *Planta* 214 562–571. 10.1007/s004250100659 11925040

[B41] Piotrowski M. Schemenewitz A. Lopukhina A. Müller A. Janowitz T. Weiler E. W. (2004). Desulfoglucosinolate sulfotransferases from *Arabidopsis thaliana* catalyze the final step in the biosynthesis of the glucosinolate core structure. *J. Biol. Chem.* 279 50717–50725. 10.1074/jbc.M407681200 15358770

[B42] Redovnikovic I. R. Glivetic T. Delonga K. Vorkapic-Furac J. (2008). Glucosinolates and their potential role in plant. *Period. Biol.* 110 297–309.

[B43] Rohr F. Ulrichs C. Mewis I. (2009). Variability of aliphatic glucosinolates in *Arabidopsis thaliana* (L.)-Impact on glucosinolate profile and insect resistance. *J. Appl. Bot. Food Qual.* 82 131–135. 17385519

[B44] Schranz E. M. Manzaneda A. J. Windsor A. J. Clauss M. J. Mitchell-Olds T. (2009). Ecological genomics of *Boechera stricta*: identification of a QTL controlling the allocation of methionine- vs. branched chain amino acid- derived glucosinolates and levels of insect herbivory. *Heredity* 102 465–474. 10.1038/hdy.2009.12 19240753 PMC2775550

[B45] Schranz M. E. Mohammadin S. Edger P. P. (2012). Ancient whole genome duplications, novelty and diversification: the WGD Radiation Lag-Time Model. *Curr. Opin. Plant Biol.* 15 147–153. 10.1016/j.pbi.2012.03.011 22480429

[B46] Sønderby I. E. Geu-Flores F. Halkier B. A. (2010). Biosynthesis of glucosinolates - gene discovery and beyond. *Trends Plant Sci.* 15 283–290. 10.1016/j.tplants.2010.02.005 20303821

[B47] Sotelo T. Soengas P. Velasco P. Rodriguez V. M. Cartea M. E. (2014). Identification of metabolic QTLs and candidate genes for glucosinolate synthesis in *Brassica oleracea* leaves, seeds flower buds. *PLoS ONE* 9:e91428 10.1371/journal.pone.0091428 PMC3948865 24614913

[B48] Van Zandt P. A. (2007). Plant defense, growth, and habitat: a comparative assessment of constitutive and induced resistance. *Ecology* 88 1984–1993. 10.1890/06-1329.1 17824430

[B49] Velasco P. Soengas P. Vilar M. Cartea M. E. del Rio M. (2008). Comparison of glucosinolate profiles in leaf and seed tissues of different *Brassica napus* crops. *J. Am. Soc. Hortic. Sci.* 133 551–558.

[B50] Velchev. (2015). “Plants,” in *Red Data Book of the PR Bulgaria* Vol. 1 ed. Velchev V. (Sofia: Publishing House Bulgarian Academy of Sciences).

[B51] Wei Z. Julkowska M. M. Laloë J. O. Hartman Y. de Boer G. J. Michelmore R. W. (2014). A mixed-model QTL analysis for salt tolerance in seedlings of crop-wild hybrids of lettuce. *Mol. Breed.* 34 1389–1400. 10.1007/s11032-014-0123-2

[B52] Windsor A. J. Reichelt M. Figuth A. Svatoš A. Kroymann J. Kliebenstein D. J. (2005). Geographic and evolutionary diversification of glucosinolates among near relatives of *Arabidopsis thaliana* (Brassicaceae). *Phytochemistry* 66 1321–1333. 10.1016/j.phytochem.2005.04.016 15913672

