# Supplementary material for: Flowering Locus C (FLC) Is a Potential Major Regulator of Glucosinolate Content across Developmental Stages of Aethionema arabicum (Brassicaceae)
Source: Front Plant Sci. 2017 May 26;8:876. doi: 10.3389/fpls.2017.00876 (PMC5445170; doi:10.3389/fpls.2017.00876)
Supplement: TABLE S2 — Significant QTLs from multi environment analyses in Aethionema arabicum TURxCYP recombinant inbred lines. [file Table_2.DOCX]

Suppl. Table 2: Significant QTLs from multi environment analyses in *Aethionema arabicum* TURxCYP recombinant inbred lines. The percentage of variability explained is shown for every tissue that had a significant (α ≤ 0.05) interaction with the QTL.

| GS | **QTL** | **Marker** | **LG^a^** | **Pos. (cM)^b^** | **-Log(p)** | **Tissue^c^** | **PVE %^d^** |
| --- | --- | --- | --- | --- | --- | --- | --- |
| 8MSOO | Q6.1  Q8.2 | S5_745413  S44_973817 | 6  8 | 16.57  150.96 | 5.79  11.73 | Seed  Fruit  Seed | 15.3  33.2*  15.4 |
| 3MSOP | Q3.1  Q6.2  Q8.2 | S17_509088  S58_8426  S44_1023171 | 3  6  8 | 87.32  72.84  149.9 | 4.01  5.36  13.69 | Fruit  Fruit  Fruit | 7.7  8.9  22.6 |
| 3MTP | Q6.2  Q8.2 | S40_63489  S44_827783 | 6  8 | 74.62  153.91 | 5.04  11.42 | Fruit  Leaf  Fruit  Leaf | 7.2  15.3  25.9*  19.6 |
| 8MSOO | Q1.1  Q8.2 | S22_10632  S44_973817 | 1  8 | 113.3  153.91 | 4.02  27.33 | Fruit  Fruit | 6.5  43.6* |
| All Aliphatic | Q1.1  Q6.2  Q8.2 | S46_1212765  S133_2358  S44_827783 | 1  6  8 | 117.43  72.89  153.91 | 5.12  5.66  23.65 | Fruit  Fruit  Fruit  Leaf | 6.8  8.3  34.8*  19.9 |
| Ratio Aliphatic/Indolic | Q1.1  Q8.2 | S22_488196  S44_827783 | 1  8 | 108.08  153.91 | 2.88  6.83 | Fruit  Fruit | 6.5  15.1 |
| All GS Compounds | Q1.1  Q6.2  Q8.2 | S46_1212765  S58_8426  S44827783 | 1  6  8 | 117.43  72.84  153.91 | 4.82  6.25  19.42 | Fruit  Fruit  Fruit  Leaf | 6.9  10  30.8*  17.7 |

^a^) Linkage Group. ^b^) Marker Position in centimorgans. ^c^) Significant tissues (α ≥ 0.05).^d^) Percentage of variance explained. *) major QTL (PVE ≥ 25%, after Burke et al 2002)
