# Supplementary figures and images for: Flowering Locus C (FLC) Is a Potential Major Regulator of Glucosinolate Content across Developmental Stages of Aethionema arabicum (Brassicaceae)
Source: Front Plant Sci. 2017 May 26;8:876. doi: 10.3389/fpls.2017.00876 (PMC5445170; doi:10.3389/fpls.2017.00876)

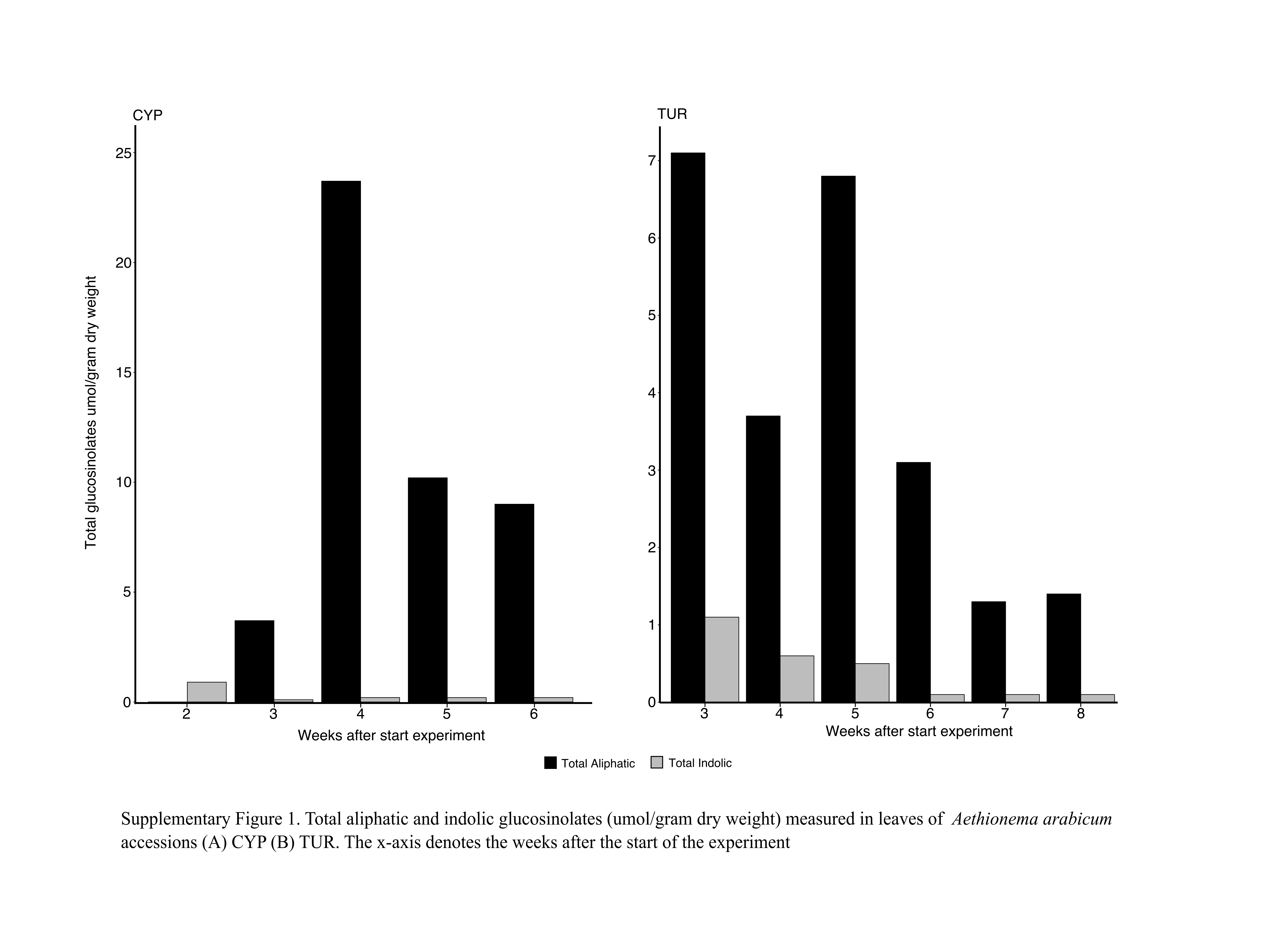

Supplement: Supplementary file 4 [file Image_1.TIFF]

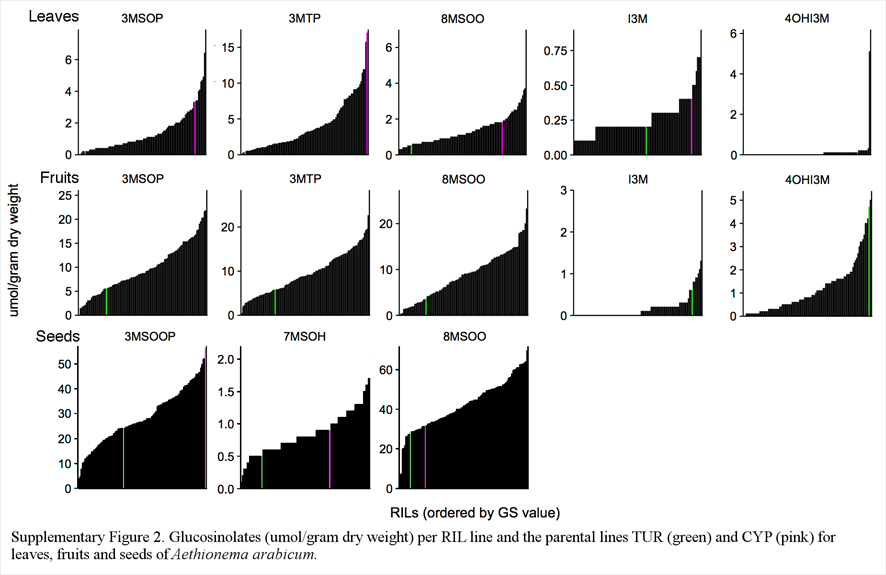

Supplement: Supplementary file 5 [file Image_2.TIF]
